# Association of the newly proposed dietary index for gut microbiota and all-cause and cardiovascular mortality among individuals with diabetes and prediabetes

**DOI:** 10.3389/fnut.2025.1621277

**Published:** 2025-08-14

**Authors:** Wenjing Song, Daoqin Liu, Zihe Xing, Luqing Jiang, Yu Tang, Zichen Xu, Lei Li, Shuai Yan, Xia Fu, Yuping Wang, Qiwen Wu

**Affiliations:** ^1^Department of Laboratory Medicine, The First Affiliated Hospital, Wannan Medical College, Wuhu, Anhui, China; ^2^Department of Nephrology, The First Affiliated Hospital, Wannan Medical College, Wuhu, Anhui, China

**Keywords:** dietary index of gut microbiota (DI-GM), diabetes, prediabetes, all-cause mortality, cardiovascular mortality

## Abstract

**Background:**

The Gut Microbiota Dietary Index (DI-GM) is a newly developed assessment tool that quantitatively evaluates the nutritional modulation of intestinal microbial communities through systematic characterization of diet-microbiome interactions. The relationship between DI-GM and the risk of death has not been elucidated in patients with diabetes or prediabetes. The present cohort study examined the longitudinal relationship between DI-GM scores and both overall mortality and mortality from cardiovascular disease in this clinically vulnerable population.

**Method:**

The investigation used data from the National Health and Nutrition Examination Survey (NHANES) 2007–2018. Analytical approaches, including multivariable-adjusted Cox proportional hazards regression, restricted cubic spline (RCS) modeling, stratified subgroup evaluations, and sensitivity assessments, were employed to examine the relationships linking DI-GM scores to mortality outcomes among individuals with diabetes or prediabetes.

**Result:**

During an average monitoring duration of 77.39 months within the cohort of 8,409 participants, 1,430 fatalities from all causes were documented, including 381 cases attributed to cardiovascular events. Multivariable-adjusted Cox regression analyses showed a negative correlation, with a 1-unit increase in DI-GM corresponding to an 8% lower all-cause mortality risk (HR = 0.92, 95% CI: 0.89–0.95; *p* < 0.001) and an 11% reduction in cardiovascular-specific mortality (HR = 0.89, 95% CI: 0.83–0.95; *p* < 0.001). When comparing extreme quartiles of DI-GM distribution, participants in the highest quartile exhibited 26% lower all-cause mortality (HR = 0.74, 95% CI: 0.63–0.87; *p* < 0.001; trend *p* < 0.05) and 30% lower cardiovascular mortality (HR = 0.70, 95% CI: 0.52–0.96; *p* = 0.025; trend *p* < 0.05) than those in the lowest quartile. Subgroup analyses confirmed the consistency of the results in most categories. Restricted cubic splines demonstrated negative correlations between DI-GM and both mortality outcomes. The Beneficial Gut Microbiota Score (BGMS) exhibited inverse associations with mortality risks, while the Unfavorable Gut Microbiota Score (UGMS) showed no significant relationship. In the sensitive analysis, the robustness of multiple interpolation results was verified by deleting missing data.

**Conclusion:**

Among patients with diabetes or prediabetes, elevated DI-GM levels were negatively correlated with all-cause mortality and cardiovascular mortality risks.

## Introduction

Diabetes is a major public health challenge worldwide. From 1990 to 2021, global diabetes prevalence increased threefold, with projections indicating over 1.3 billion affected individuals by 2025 if uncontrolled (1). According to diagnostic criteria established by the American Diabetes Association (ADA), diabetes mellitus is classified as a persistent metabolic disorder that is diagnostically confirmed through sustained hyperglycemia (defined as fasting plasma glucose ≥ 7.0 mmol/L) or elevated glycosylated hemoglobin (HbA1c) levels ≥ 6.5%, pathophysiologically rooted in a disorder of insulin production or cellular responsiveness (2). Prediabetes, a transitional phase between normal glucose regulation and diabetes, affects approximately 720 million people globally (3, 4). Cardiovascular disease, which is strongly linked to diabetes, contributes to over 50% of mortality in this population (5). Epidemiological investigations have consistently established that individuals with diabetes or prediabetes have significantly heightened risks for both all-cause mortality and cardiovascular-specific fatality outcomes (6–8).

Gut microbial composition and functionality exert critical influences on diabetes pathogenesis (9, 10). The gut microbiome can influence systemic inflammation, insulin sensitivity and energy balance through various mechanisms, such as the fermentation of dietary fiber, the production of short-chain fatty acids (SCFA), and the regulation of gut-brain signaling (11–13). A substantial corpus of research has demonstrated that individuals with diabetes or prediabetes have a reduced abundance and functional capacity of bacteria which produce butyrate (14). Dietary factors, particularly fiber intake, strongly regulate butyrate-producing microbial populations, potentially enhancing glycemic control (15). Nutritional patterns are the primary determinants of gut microbiome structure (15). Evidence indicates that dietary-derived microbial metabolites positively modulate insulin secretion, sensitivity, and diabetes incidence (16–19). Dietary modifications alter host microbial communities, cardiometabolic profiles, and immune responses through interconnected pathways (20). Epidemiological studies have confirmed that a nutritionally imbalanced dietary structure characterized by excessive intake of red meat and processed meat products, alcohol abuse, frequent consumption of refined carbohydrates (such as white bread), and sugar-added beverages has a significant dose-dependent association with the prediabetes state and the risk of diagnosed diabetes (21, 22).

Many studies have examined the effects of individual dietary components and their impact on gut microbiota (23, 24). According to Kase et al., the DI-GM was developed based on a large number of literature reviews and indicated that DI-GM is related to indirect biomarkers of intestinal microbiota diversity (25). The DI-GM aims to quantify the effects of nutritional patterns on the structure and metabolic characteristics of the gut microbiome by systematically evaluating the interaction between diet and microbiome. Based on a large number of review articles, 14 types of foods and nutrients have been determined to have beneficial or adverse effects on the gut microbiota. Compared with HEI-2015 and the Mediterranean Diet Score (MDS), the DI-GM focuses on a broader range of gut microbiome attributes (for example, the production of short-chain fatty acids (SCFA), changes in microbiota phyla, and specific bacterial species), providing a more comprehensive assessment of diet-microbiome relationships (25, 26). Malesza et al.‘s research indicates that a high-fat diet is associated with reduced microbial richness and increased intestinal permeability (27). A high-fat diet rich in meat protein may promote different and less diverse populations of sulfur-metabolizing bacteria (28). Recent studies have shown a negative relationship between DI-GM and the risk of developing diabetes (29). In addition, the incidence of other diseases associated with DI-GM, such as stroke (30), chronic kidney disease (31), and depression (32), is negatively correlated. Despite growing evidence on gut microbiota-diet interactions, the longitudinal associations linking the DI-GM to mortality risk in individuals with diabetes or prediabetes have not been fully established. The purpose of our study was to systematically evaluate the prognostic value of DI-GM for all-cause mortality and cardiovascular-specific mortality endpoints in a high-risk population using the NHANES data system.

## Method

### Population

**Figure 1:**
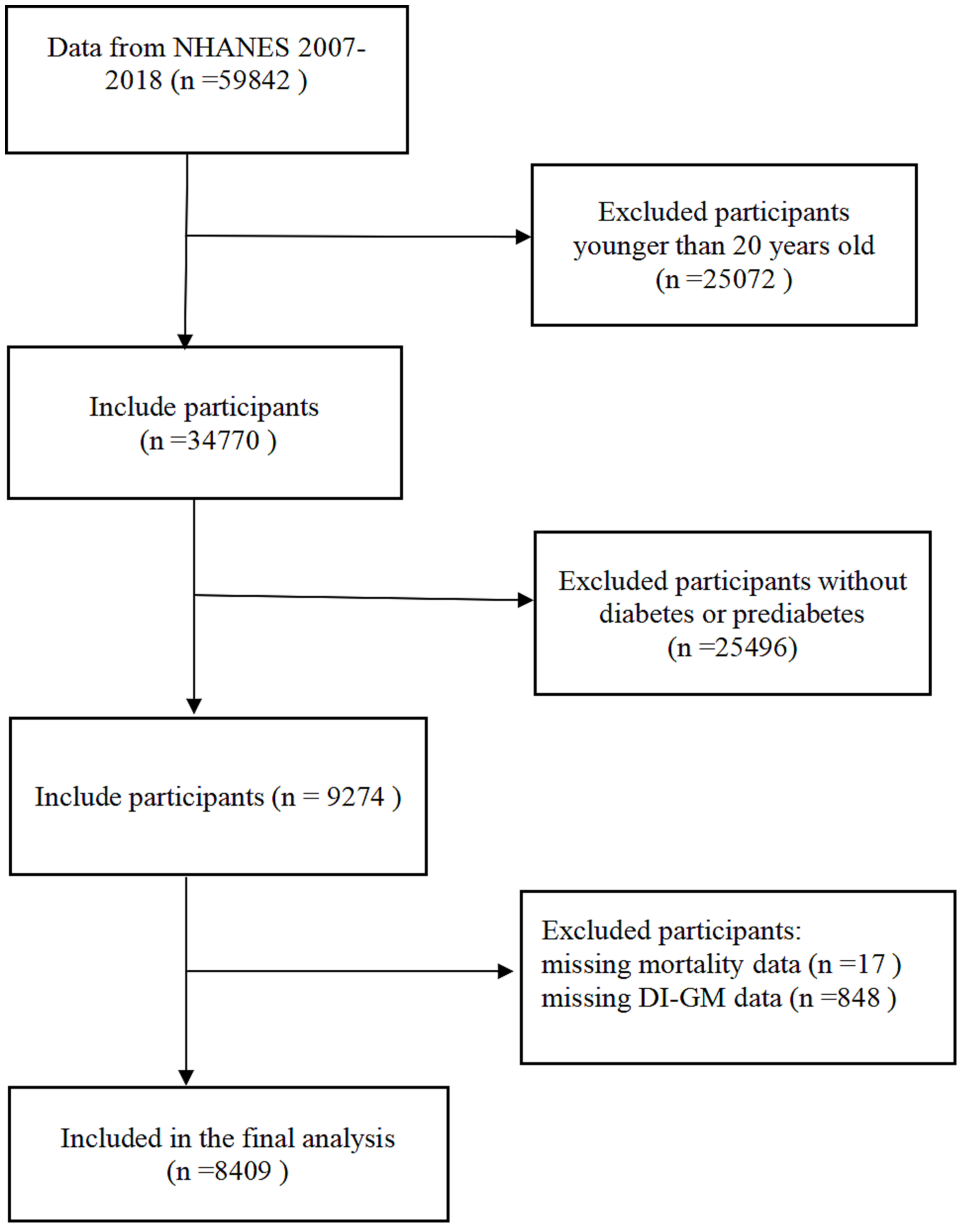
Screening process of participants.

The investigation used data from the NHANES. The survey’s core mission focuses on tracking health status and nutritional parameters among non-institutionalized civilian residents of the United States through three-tiered data collection: standardized in-home questionnaires, comprehensive physical examinations at Mobile Examination Centers (MECs), and biochemical assays conducted in certified laboratories, all implemented via a complex multistage probability cluster sampling design. All study protocols received ethical approval from the NCHS Institutional Review Board, with written informed consent secured from each participant prior to data collection. The analytical cohort initially comprised 59,842 individuals drawn from six consecutive NHANES cycles spanning 2007–2018, with data retrieved from the official NHANES repository. We excluded the following individuals: (1) age <20 years at baseline (*n* = 25,072); (2) absence of confirmed diabetes or prediabetes diagnosis (*n* = 25,496); (3) incomplete mortality follow-up (*n* = 17); (4) lack of complete DI-GM measurements (*n* = 848). The final analytical cohort comprised 8,409 eligible individuals (Figure 1).

### Definition of diabetes and prediabetes

Diabetes diagnosis was determined by meeting ≥1 of these parameters: (1) elevated fasting plasma glucose (FPG ≥ 7.0 mmol/L) or 2-h oral glucose tolerance test (2 h-OGTT) ≥ 11.1 mmol/L; (2) glycated hemoglobin (HbA1c) ≥ 6.5%; (3) random blood glucose ≥ 11.1 mmol/L; (4) your doctor has diagnosed you with diabetes or you are currently using insulin.

Prediabetes classification required fulfillment of ≥ 1 criterion: (1) 2 h-OGTT results 7.8–11.0 mmol/L; (2) HbA1c 5.7–6.4%; (3) FPG 6.1–6.9 mmol/L; (4) a physician’s diagnosis of prediabetes.

### Dietary index of gut microbiota

The Dietary Index of Gut Microbiota (DI-GM) was developed to evaluate how diet influences the composition of the gut microbiome through literature-derived parameters (25). The index was derived from two 24-h dietary recall datasets, with the average value of these datasets being calculated. The initial data pertaining to the recall were collated at the MEC, while subsequent data were obtained through the medium of telephone interviews. To ensure data quality, the NHANES implemented standardized interviewer training protocols with validated assessment tools, effectively minimizing measurement errors related to recall bias and interviewer variability. The DI-GM scoring system evaluates 14 dietary components categorized into two groups: health-promoting constituents (fermented dairy products, soybeans, chickpeas, fiber, cranberries, avocados, broccoli, coffee, tea, and whole grains,) and detrimental elements (refined cereals, unprocessed/processed red meats, and high-fat diets [≥ 40% total energy intake]). Specific tea varieties were excluded due to insufficient granularity in NHANES dietary records. Scoring criteria were defined as follows: beneficial components received 1 point when intake met or exceeded sex-stratified median values, otherwise 0; detrimental components scored 1 point when consumption fell below sex-specific medians (or < 40% energy from fat for high-fat diets), otherwise 0. The composite DI-GM scale ranges from 0–13 points and comprising two subscales: beneficial (0–9) and detrimental (0–4) components. Higher aggregate scores reflected stronger microbiota-friendly dietary profiles. For analytical purposes, the participants were stratified into quartiles based on DI-GM scores. The complete scoring criteria and food classifications are provided in Supplementary Table 1.

### Covariant

The covariates considered in the present study included age, gender, race/ethnicity, educational attainment, marital status, family poverty income ratio (PIR), body mass index (BMI), smoking, alcohol consumption, hypertension, physical activity, coronary heart disease, stroke, cancer, insulin usage status and total cholesterol. Educational attainment was categorized into three categories: below high school, high school, and beyond high school. The classification of ethnicity included the categories Mexican-American, other Hispanic, non-Hispanic Asian, non-Hispanic White people, non-Hispanic Black, and other races. Marital status was dichotomized into married/cohabiting partnerships and unmarried status (encompassing widowed, separated, and divorced individuals). Body mass index (BMI) was stratified according to WHO criteria: normal weight (18.5–24.9 kg/m^2^), overweight (25.0–29.9 kg/m^2^), and obese (≥ 30.0 kg/m^2^). Poverty-income ratios were categorized into three tiers: ≤ 1.0 (below the poverty line), 1.1–3.0 (low-moderate income), and ≥ 3.1 (higher socioeconomic status). Smoking was categorized into three categories: individuals who have never smoked, those who used to smoke but have quit smoking, and current smokers. Alcohol consumption patterns were operationalized as: abstainers (< 12 lifetime drinks or ≥12 drinks without past-year consumption), low to moderate drinkers (women ≤ 1 drink/day; men ≤ 2 drinks/day), and heavy drinkers (women > 1 drink/day; men > 2 drinks/day). Clinically confirmed hypertension was defined as self-reported physician diagnosis. According to previous studies, physical activity is divided into low physical activity (<500 MET/wk) and high physical activity (≥ 500 MET/wk) (33). The insulin usage status was categorized as either using or not using. Coronary heart disease: Being informed that one has coronary heart disease. Stroke: Being informed that one has had a stroke. Cancer: Being informed that one has had cancer or a malignant tumor.

### Mortality assessment

Mortality outcomes were ascertained through linkage with the National Death Index (NDI), a federal mortality registry maintained by the National Center for Health Statistics (NCHS). The observational window spanned from study enrollment through December 31, 2019, capturing complete mortality surveillance data^1^. Cause-specific mortality classifications adhered to ICD-10 coding protocols. All-cause mortality aggregated all documented fatalities, including but not limited to cardiovascular pathologies (ICD-10 I00-I78), neoplastic disorders (C00-C97), accidental trauma (V01-X59), cerebrovascular events (I60-I69), diabetes-related complications (E10-E14), and other etiologies. Cardiovascular mortality specifically included fatal diseases of the cardiovascular system. The follow-up duration was determined as the period between baseline evaluation and either mortality occurrence or study termination (December 31, 2019).

### Statistical analysis

Statistical analyses were performed using R software version 4.3.0 and SPSS (IBM) version 27. The participant characteristics were stratified according to the DI-GM quartiles. Numerical variables are expressed as mean ± standard deviation, and categorical data are expressed as proportion (%) and frequency distribution. For continuous measurements, the nonparametric Kruskal-Wallis test was used for comparative analysis, and for inter-group frequency comparison, the Pearson χ^2^ test was used. Three progressively adjusted Cox proportional hazards models were constructed to assess the relationship between DI-GM and death outcomes. Model 1: Crude model without covariates; Model 2: Demographic-adjusted model controlling for age, gender, and race/ethnicity; Model 3: Multivariable-adjusted model incorporating socioeconomic (education attainment, marital status, poverty-income ratio) and lifestyle factors (BMI, smoking, alcohol consumption, physical activity) alongside clinical variables (hypertension, coronary heart disease, stroke, cancer, insulin usage status, total cholesterol). For missing values, we adopt a multiple interpolation method based on the chain equation of the Bayesian framework. Nonlinear associations between DI-GM scores and mortality endpoints were evaluated using restricted cubic splines (RCS) with three prespecified knots, modeled within the multivariable-adjusted Cox regression framework. Stratified analyses across clinically relevant subgroups were conducted using interaction term assessments to detect potential effect modifications. Sensitivity evaluations were performed by reanalyzing complete-case datasets (excluding observations with missing covariates), thereby confirming the robustness of primary findings against missing data assumptions. Statistically significant was a *p* value < 0.05.

## Result

### Participant baseline characteristics

In this study, a total of 8,409 participants were included, with a mean age of 59.32 years (±14.76). The vast majority of all participants were non-Hispanic White people, of whom 51.83% were male (Table 1). Compared to the lowest group (Q1), groups with older ages, higher numbers of non-Hispanic White people, and higher education levels were concentrated in the higher DI-GM groups. However, we also observed a significant decrease in obesity, smoking, and heavy drinking rates with the increase of DI-GM, indicating a potential association between healthy living and elevated DI-GM. In the DI-GM group, there was no statistically significance differences in gender, marital status, physical activity, prevalence of hypertension, prevalence of coronary heart disease, prevalence of stroke, and insulin usage status.

**Table 1:** Participant baseline characteristics.

Variable	DI-GM				*p*
	Q1	Q2	Q3	Q4	
Age, years, mean (SD)	56.32 ± 15.34	57.70 ± 14.99	59.00 ± 14.95	62.07 ± 13.68	<0.001
TC, mean (SD)	4.88 ± 1.14	4.96 ± 1.22	4.93 ± 1.20	4.88 ± 1.17	0.065
Gender, *n* (%)					0.061
Male	735 (51.83)	1,037 (53.12)	1,124 (53.27)	1,462 (49.91)	
Female	683 (48.17)	915 (46.88)	986 (46.73)	1,467 (50.09)	
Race, *n* (%)					<0.001
Mexican-American	223 (15.73)	398 (20.39)	419 (19.86)	443 (15.12)	
Other Hispanic	151 (10.65)	224 (11.48)	236 (11.18)	338 (11.54)	
Non-Hispanic White people	521 (36.74)	671 (34.38)	828 (39.24)	1,246 (42.54)	
Non-Hispanic Black	397 (28.00)	486 (24.90)	447 (21.18)	536 (18.30)	
Other races	126 (8.89)	173 (8.86)	180 (8.53)	366 (12.50)	
Education, *n* (%)					<0.001
Below high school	457 (32.23)	661 (33.86)	736 (34.88)	845 (28.85)	
High school	373 (26.30)	467 (23.92)	483 (22.89)	649 (22.16)	
Above high school	588 (41.47)	824 (42.21)	891 (42.23)	1,435 (48.99)	
Marital, *n* (%)					0.989
No spouse/partner/divorced/single	564 (39.77)	776 (39.75)	837 (39.67)	1,175 (40.12)	
Married/cohabiting	854 (60.23)	1,176 (60.25)	1,273 (60.33)	1754 (59.88)	
Smoking, *n* (%)					<0.001
Do not smoke	705 (49.72)	960 (49.18)	1,052 (49.86)	1,573 (53.70)	
Smoking	415 (29.27)	601 (30.79)	696 (32.99)	977 (33.36)	
Now smoking	298 (21.02)	391 (20.03)	362 (17.16)	379 (12.94)	
Hypertension, *n* (%)					0.212
No	478 (33.71)	664 (34.02)	745 (35.31)	951 (32.47)	
Yes	940 (66.29)	1,288 (65.98)	1,365 (64.69)	1978 (67.53)	
BMI, *n* (%)					<0.001
Normal <25	189 (13.33)	289 (14.81)	325 (15.40)	526 (17.96)	
0verweight 25–30.0	368 (25.95)	586 (30.02)	624 (29.57)	949 (32.40)	
Obesity> 30	861 (60.72)	1,077 (55.17)	1,161 (55.02)	1,454 (49.64)	
Drinking, *n* (%)					<0.001
No alcohol	297 (20.94)	397 (20.34)	471 (22.32)	612 (20.89)	
Low/moderate alcohol consumption	703 (49.58)	1,014 (51.95)	1,115 (52.84)	1707 (58.28)	
Heavy drinking	418 (29.48)	541 (27.72)	524 (24.83)	610 (20.83)	
PIR, *n* (%)					<0.001
≤1.00	374 (26.38)	466 (23.87)	530 (25.12)	544 (18.57)	
1.00–3.00	665 (46.90)	915 (46.88)	979 (46.40)	1,263 (43.12)	
≥3.00	379 (26.73)	571 (29.25)	601 (28.48)	1,122 (38.31)	
Physical activity, *n* (%)					0.095
Low physical activity	1,009 (71.16)	1,353 (69.31)	1,528 (72.42)	2,117 (72.28)	
High physical activity	409 (28.84)	599 (30.69)	582 (27.58)	812 (27.72)	
Coronary heart disease, *n* (%)					0.226
Yes	111 (7.83)	150 (7.68)	186 (8.82)	268 (9.15)	
No	1,307 (92.17)	1802 (92.32)	1924 (91.18)	2,661 (90.85)	
Stroke, *n* (%)					0.629
Yes	97 (6.84)	149 (7.63)	154 (7.30)	197 (6.73)	
No	1,321 (93.16)	1803 (92.37)	1956 (92.70)	2,732 (93.27)	
Cancer, *n* (%)					0.002
Yes	184 (12.98)	242 (12.40)	273 (12.94)	463 (15.81)	
No	1,234 (87.02)	1710 (87.60)	1837 (87.06)	2,466 (84.19)	
Insulin usage status, *n* (%)					0.167
Using	196 (13.82)	280 (14.34)	310 (14.69)	371 (12.67)	
Not using	1,222 (86.18)	1,672 (85.66)	1800 (85.31)	2,558 (87.33)	
DM, *n* (%)					0.401
Diabetes	988 (69.68)	1,400 (71.72)	1,481 (70.19)	2037 (69.55)	
Prediabetes	430 (30.32)	552 (28.28)	629 (29.81)	892 (30.45)	

Numerical variables were summarized using mean ± SD, with statistical comparisons employing nonparametric Kruskal-Wallis tests. Categorical variables were presented as counts (percentages) analyzed through Pearson’s χ^2^ tests.

### Correlation between DI-GM and all-cause and cardiovascular mortality

During the average 77.39-month follow-up, there were 1,430 all-cause deaths in 8409 participants, including 381 deaths due to cardiovascular disease. Multivariable-adjusted Cox proportional hazards regression models were used to assess the mortality risk associations with DI-GM scores. (Table 2) In Model 1 (unadjusted model), DI-GM was not significantly associated with all-cause mortality and cardiovascular mortality (*p* > 0.05). In Model 2 (adjusted for demographic factors), each 1-unit increment in DI-GM levels corresponded to a 9% diminished risk of all-cause mortality (HR = 0.91, 95% CI 0.88–0.94) and 13% decreased likelihood of cardiovascular-specific mortality (HR = 0.87, 95% CI 0.82–0.94), both meeting conventional statistical significance (*p* < 0.001). Participants in Q4 showed a 30% lower hazard ratio for all-cause mortality than those in Q1 (HR = 0.70, 95% CI 0.60–0.82; trend *p* < 0.001). Similarly, the Q4 group demonstrated a 35% reduced risk of cardiovascular mortality relative to the Q1 group (HR = 0.65, 95% CI 0.48–0.89, trend *p* < 0.001). In Model 3 (the fully adjusted model), per 1-unit increment in DI-GM concentration, multivariable analysis revealed statistically significant risk attenuation: all-cause mortality showed an 8% diminished hazard (HR = 0.92, 95% CI 0.89–0.95), while cardiovascular death exhibited an 11% decreased likelihood (HR = 0.89, 95% CI 0.83–0.95), both with *p* < 0.001. Participants in Q4 demonstrated a 26% lower all-cause mortality hazard relative to their Q1 counterparts (HR = 0.74, 95% CI 0.63–0.87; trend *p* < 0.05), with cardiovascular mortality showing comparable risk reduction (HR = 0.70, 95% CI 0.52–0.96; trend *p* < 0.05). In the complete model that underwent full adjustment, BGMS demonstrated a remarkably negative correlation with both all-cause mortality risk and cardiovascular mortality risk (*p* < 0.001). Conversely, UGMS was no significantly association with either all-cause mortality or cardiovascular mortality (*p* > 0.05).

**Table 2:** COX regression model for the connection between DI-GM and death.

Variable	Model 1		Model 2		Model 3	
	HR (95%CI)	*p-*value	HR (95%CI)	*p*-value	HR (95%CI)	*p-*value
All-cause death						
DI-GM	0.99 (0.96,1.03)	0.698	0.91 (0.88,0.94)	<0.001	0.92 (0.89,0.95)	<0.001
DI-GM (Quartile)						
Q 1	1.00 (Reference)		1.00 (Reference)		1.00 (Reference)	
Q 2	0.98 (0.83,1.16)	0.822	0.93 (0.79,1.10)	0.418	0.95 (0.80,1.13)	0.548
Q 3	0.96 (0.82,1.14)	0.666	0.82 (0.69,0.96)	0.016	0.80 (0.68,0.94)	0.008
Q 4	0.99 (0.85,1.16)	0.945	0.70 (0.60,0.82)	<0.001	0.74 (0.63,0.87)	<0.001
*P* for Trend	0.987		<0.001		<0.001	
BGMS	0.94 (0.90,0.98)	0.003	0.86 (0.82,0.90)	<0.001	0.89 (0.85,0.93)	<0.001
UGMS	1.07 (1.02,1.13)	0.004	0.99 (0.94,1.04)	0.756	0.98 (0.93,1.03)	0.400
Cardiovascular death						
DI-GM	0.96 (0.90,1.03)	0.225	0.87 (0.82,0.94)	<0.001	0.89 (0.83,0.95)	<0.001
DI-GM (Quartile)						
Q 1	1.00 (Reference)		1.00 (Reference)		1.00 (Reference)	
Q 2	1.10 (0.80,1.52)	0.547	1.05 (0.76,1.45)	0.762	1.10 (0.80,1.51)	0.567
Q 3	0.92 (0.66,1.27)	0.599	0.77 (0.56,1.07)	0.121	0.75 (0.54,1.04)	0.087
Q 4	0.93 (0.69,1.26)	0.658	0.65 (0.48,0.89)	0.006	0.70 (0.52,0.96)	0.025
*P* for Trend	0.351		<0.001		0.001	
BGMS	0.89 (0.82,0.97)	0.010	0.82 (0.75,0.89)	<0.001	0.86 (0.78,0.94)	<0.001
UGMS	1.06 (0.96,1.17)	0.225	0.98 (0.89,1.08)	0.629	0.95 (0.86,1.05)	0.347

HR: Risk Ratio, CI: Confidence Interval. Model 1, unadjusted; Model 2, adjusted according to age, gender and race; Model 3, adjusted according to age, gender, race, educational attainment, marital status, BMI, PIR, hypertension, drinking, smoking, physical activity, coronary heart disease, stroke, cancer, insulin use status and total cholesterol.

### Detection of nonlinear outcomes between DI-GM and mortality

**Figure 2:**
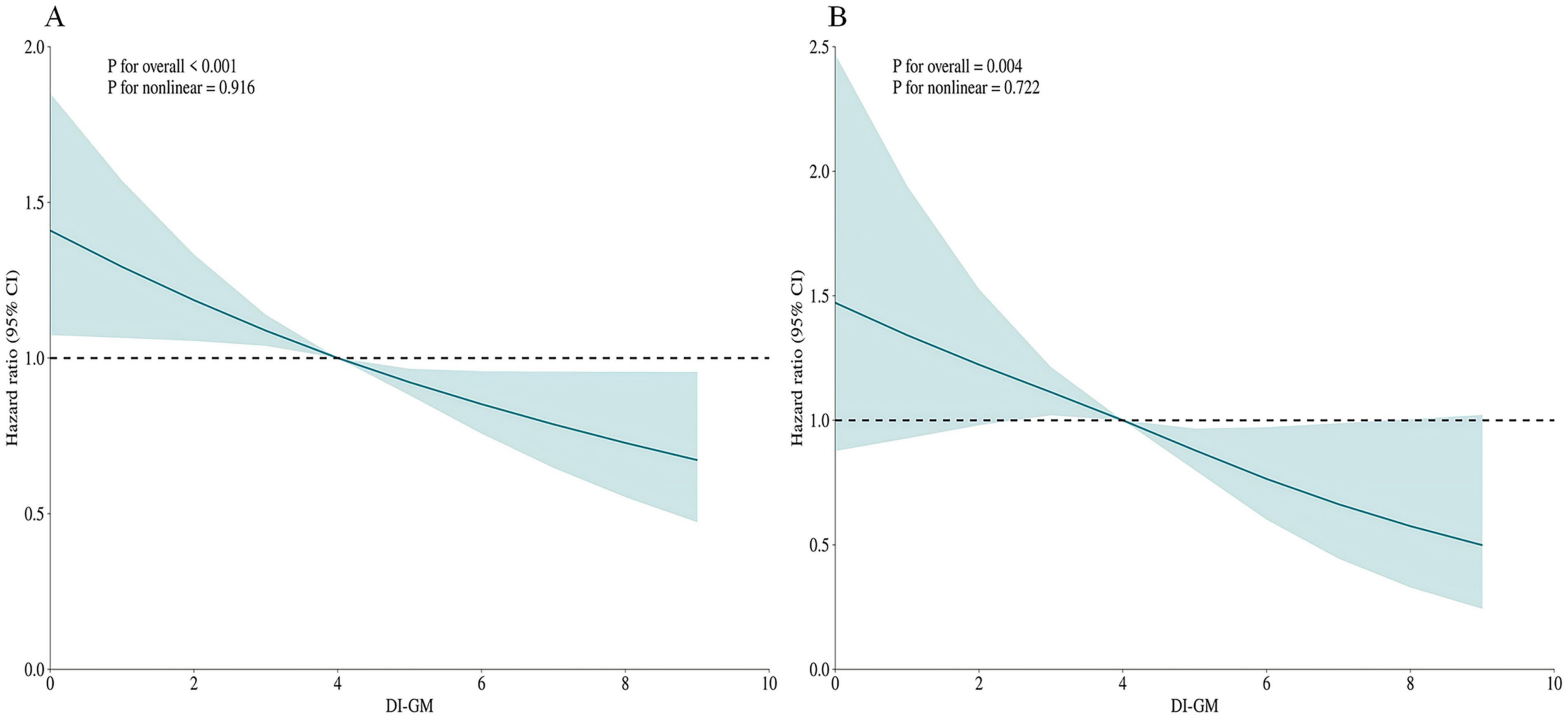
The restricted cubic spline (RCS) curves for all-cause mortality **(A)** and cardiovascular mortality **(B)** in patients with diabetes or prediabetes, adjusted for age, gender, race, educational attainment, marital status, BMI, PIR, hypertension, drinking, smoking, physical activity, coronary heart disease, stroke, cancer, insulin use status and total cholesterol.

**Figure 3:**
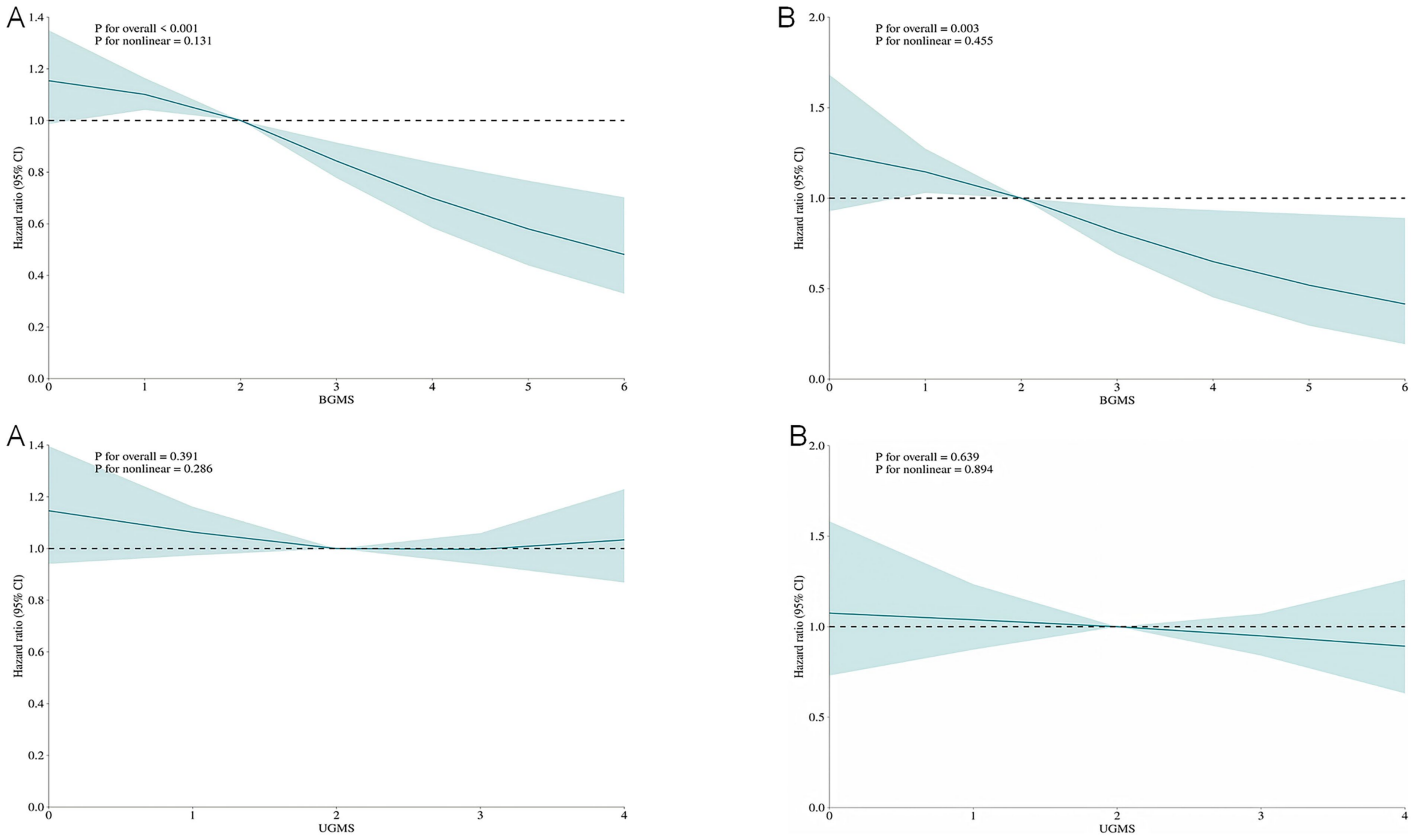
The restricted cubic spline (RCS) curves for all-cause mortality **(A)** and cardiovascular mortality **(B)** among patients with diabetes or prediabetes, comparing Beneficial Gut Microbiota Score (BGMS) and Unfavorable Gut Microbiota Score (UGMS). The model was adjusted for age, gender, race, educational attainment, marital status, BMI, PIR, hypertension, drinking, smoking, physical activity, coronary heart disease, stroke, cancer, insulin use status and total cholesterol.

To assess potential non-linear associations, restricted cubic spline (RCS) modeling was implemented within the fully adjusted Cox proportional hazards framework (Model 3). (Figure 2) The increase in the DI-GM score was significantly and negatively correlated with the risk of all-cause mortality and cardiovascular mortality, with a primarily linear correlation (non-linear test *p* > 0.05). A linear negative correlation was also observed between the BGMS and all-cause and cardiovascular mortality (non-linear test *p* > 0.05), whereas no significant relationship was found between the UGMS and the two mortality rates. (Figure 3).

### Subgroup analysis

**Figure 4:**
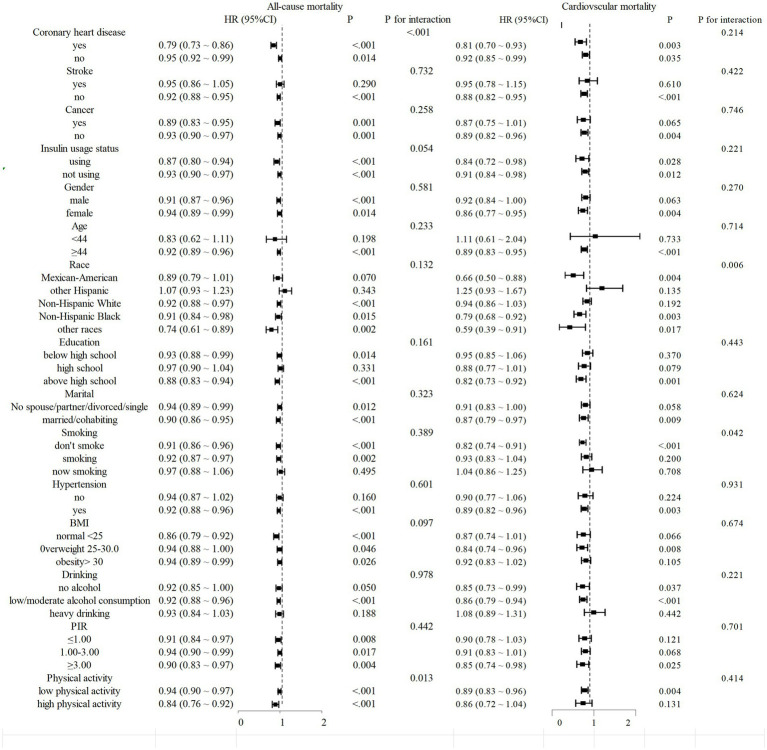
Stratified analysis of mortality from diabetes or prediabetes with DI-GM. Stratified multivariable analyses were performed using Model 3, with all stratification variables systematically excluded from covariate adjustment to avoid over-adjustment bias. This methodological approach ensured that variables used for subgroup categorization were not included as confounders in respective stratified models.

We conducted a stratified analysis to evaluate the association between different populations and mortality rates. The findings revealed that DI-GM exhibited a consistently negative correlation with mortality across most subgroups, which was statistically significant. Furthermore, in terms of cardiovascular mortality, smoking status and race interact with DI-GM, indicating potential regulatory effects (interaction *p* < 0.05). (Figure 4).

### Sensibility analysis

In the sensitivity analysis, 6,839 participants were included after excluding those who lacked covariate values when the baseline was excluded (Supplementary Table 2). The relationship between DI-GM and all-cause mortality and cardiovascular mortality demonstrated stability. Similar stability was observed in the associations between BGMS and UGMS and all-cause mortality and cardiovascular mortality.

## Discussion

This study is pioneering in its use of the substantial sample size of the NHANES database to appraise the connection between DI-GM and survival outcomes among people with diabetes or prediabetes. Our analysis revealed an inverse dose–response relationship between DI-GM scores and the risks of all-cause and cardiovascular mortality. This association was found to be highly consistent, suggesting that relatively high DI-GM scores are linked to a lower likelihood of both types of mortality. These results underscore the clinical significance of dietary patterns that enhance microbial diversity in the context of diabetes management and mortality reduction.

Insulin resistance is a critical factor in the development and progression of diabetes and its precursor states (34, 35). Individuals with lower gut microbiome diversity tended to show more significant overall obesity and insulin resistance characteristics (36). An imbalance in intestinal flora may lead to a reduction in the number of beneficial bacteria that produce short-chain fatty acids (SCFAs), especially butyric acid, which is directly related to decreased insulin sensitivity and abnormal blood sugar control (37–39). A plethora of studies have demonstrated that the diversity of the gut microbiome plays a crucial role in maintaining optimal insulin sensitivity and mitigating insulin resistance (40). An imbalance in the gut microbiome can lead to the proliferation of harmful bacteria, such as proteobacteria, which transform choline and carnitine from food into trimethylamine (TMA). Once produced, TMA is further metabolized in the liver to form trimethylamine-N-oxide (TMAO), a compound that substantially elevates the risk of cardiovascular disease by facilitating atherosclerosis, thrombosis, and inflammatory processes (41). In addition, the metabolite of the gut microbiome, TMAO, is positively correlated with all-cause mortality and cardiovascular mortality (42). A positive association has been demonstrated between elevated TMAO levels and the increased probability of developing diabetes (43). Studies have shown a connection between dietary changes and shifts in the gut microbiome (37, 44).

Intestinal diols and lactones in urine are indirect biomarkers of intestinal microbiota diversity. DI-GM was positively correlated with the levels of intestinal diols and lactones in urine, indicating enhanced diversity of the intestinal microbiota. A higher DI-GM score indicates enhanced intestinal microbiota diversity (25). Recently, Wu et al. conducted a cross-sectional study of 21,640 people in the United States and found that higher DI-GM scores were significantly associated with a reduced risk of diabetes (29). However, there is still a scarcity of longitudinal studies that have investigated the issue of mortality in patients diagnosed with diabetes mellitus or prediabetes. According to our results, a pronounced inverse association was observed between DI-GM levels and mortality outcomes, with both all-cause and cardiovascular death risks demonstrating marked reductions (*p* < 0.05). This suggests that DI-GM is associated with a reduced risk of death in patients with diabetes or prediabetes. The results in RCS demonstrated a negative linear correlation between DI-GM and BGMS for all-cause and cardiovascular mortality (non-linear *p* > 0.05). Our research results also indicate that UGMS is negatively correlated with all-cause mortality and cardiovascular mortality in patients with diabetes or prediabetes, but the difference was no statistically significant (*p* > 0.05). Tindall and Shreiner et al. demonstrated that UGMS focuses on components such as red meat and refined grains, which generate uremic toxins such as TMAO through intestinal flora metabolism, activate the NLRP3 inflammatory body, and induce endothelial cell apoptosis (45, 46). In addition, a diet high in fat and sugar but low in fiber, fruits and vegetables is associated with chronic inflammation (47). In our study, there was no significant association between UGMS and mortality. This might be due to the shorter course of diabetes in the high-exposure group of UGMS in the sample, which led to the cumulative effect of chronic inflammation not being fully manifested. Dietary optimization has demonstrated efficacy in fostering the proliferation of beneficial gut microbiota, thereby reducing the risk of death, consistent with the findings of previous studies. The absence of statistical significance in the coarse model may indicate the influence of confounding factors, particularly age. Interestingly, we performed a stratified analysis and discovered that DI-GM exerted a more pronounced effect on the mortality risk among middle-aged and elderly individuals, which might be attributable to age-related decline in metabolic reserves. Zhang et al. discovered that an augmentation in the score of DI-GM was correlated with the risk of developing sarcopenia, a condition that was prevalent among middle-aged and elderly populations and is connected with inadequate metabolic reserves (48). A substantial body of research has demonstrated that the gut microbiota plays a pivotal role in the aging process. Higher DI-GM scores have been found to be associated with a reduced risk of accelerated aging (49, 50). Our results showed a significant interaction between smoking and race in the subgroup analysis. Research indicates that smoking significantly increases the risk of all-cause and cardiovascular mortality in individuals with diabetes. However, smoking cessation is associated with a lower risk and a strong positive correlation with other cardiovascular health outcomes (including heart failure and peripheral artery disease) (51–53). Therefore, it may mask the protective factors of DI-GM, leading to an increased risk of death. The prevalence of diabetes also varies by race, with the highest prevalence among Hispanics/Latinos (54, 55).

DI-GM has many potential applications in clinical practice. By quantifying the impact of diet on the gut microbiota, DI-GM can identify high-risk groups for diabetes, cardiovascular diseases and cancer (56–58). Based on the beneficial and unfavorable components of DI-GM, dietary plans are customized for patients, such as guiding prediabetic patients to adjust the proportion of dietary fiber to reduce the risk of progression (32, 35, 57, 59).

This investigation presents several notable strengths. First, as a novel dietary evaluation metric, DI-GM has been examined for its associations with mortality in populations with diabetes or prediabetes for the first time, to our knowledge. Second, the nationally representative NHANES cohort employed a multistage probability cluster sampling design, ensuring methodological rigor and population generalizability. Third, comprehensive covariate adjustments minimized confounding biases, with sensitivity analyses confirming result robustness. Several limitations warrant consideration. First, inherent methodological constraints exist in DI-GM’s current formulation, particularly regarding the inclusion criteria for emerging dietary components requiring empirical validation. Second, our analysis lacked granular data on food preparation methods, potentially influencing microbiome interactions. Third, NHANES dietary recalls omitted tea subtype specifications, possibly affecting DI-GM scoring accuracy. Future investigations should prioritize validating disputed dietary-microbiome interactions, and elucidating microbiota-mediated dietary influences on metabolic outcomes.

## Conclusion

The research discovered that the DI-GM score exhibits an inverse relationship with both all-cause mortality and cardiovascular mortality among individuals with diabetes or pre-diabetes. A greater DI-GM score has been found to correlate with enhanced diversity of the gut microbiota, which is associated with a lower risk of mortality. These results highlight the importance of consuming foods that are beneficial to the gut microbiota in the prevention and control of diabetes and its early stages.

## Data Availability

The original contributions presented in the study are included in the article/Supplementary material, further inquiries can be directed to the corresponding author/s.

## Footnotes

^1^ https://www.cdc.gov/nchs/data-linkage/mortality-public.htm
