# Supplementary material for: Association of the newly proposed dietary index for gut microbiota and all-cause and cardiovascular mortality among individuals with diabetes and prediabetes
Source: Front Nutr. 2025 Aug 14;12:1621277. doi: 10.3389/fnut.2025.1621277 (PMC12390802; doi:10.3389/fnut.2025.1621277)
Supplement: Supplementary file 1 [file Table_1.docx]

Supplementary Table 1 Components and scoring criteria of DI-GM in NHANES

| Components of DI-GM | Included Foods within the Component | Scoring criteria |
| --- | --- | --- |
| Beneficial Gut Microbiota Score | Avocados | For each component, a score of 1 if consumption At or above the sex-specific median, else 0 |
|  | Broccoli |  |
|  | Chickpeas |  |
|  | Coffee |  |
|  | Cranberries |  |
|  | Fermented dairy (including yogurt, cheese,kefir, sour cream, buttermilk) |  |
|  | Fiber |  |
|  | Soybean (including Soymilk,Tofu) |  |
|  | Whole grains (grains defined as whole grains, containing the entire grain kernel—the bran, germ, and endosperm) |  |
|  | Green tea |  |
| Unfavorable Gut Microbiota Score | High-fat diet (% energy) | 0 if consumption at or above 40% energy from fat, else 1 For each remaining component, a score of 0 if consumption at or above the sex-specific median, else 1 |
|  | Processed meat (including frankfurters, sausages, corned beef, and luncheon meat that are made from beef, pork, or poultry) |  |
|  | Red meat (including beef, veal, pork, lamb, and game meat; excludes organ meat and cured meat) |  |
|  | Refined grains (refined grains that do not contain all of the components of the entire grain kernel) |  |
| Abbreviations: DI-GM, Dietary Index for Gut Microbiota; NHANES, National Health and Nutrition Examination Survey. | | |
