# Supplementary material for: Association of the newly proposed dietary index for gut microbiota and all-cause and cardiovascular mortality among individuals with diabetes and prediabetes
Source: Front Nutr. 2025 Aug 14;12:1621277. doi: 10.3389/fnut.2025.1621277 (PMC12390802; doi:10.3389/fnut.2025.1621277)
Supplement: Supplementary file 2 [file Table_2.docx]

Supplementary Table 2 After excluding participants with missing covariates, mortality HR (95%CI) according to DI-GM quartiles

|  | Model 1 | | Model 2 | | Model 3 | |
| --- | --- | --- | --- | --- | --- | --- |
|  | HR (95%CI) | P value | HR (95%CI) | P value | HR (95%CI) | P value |
| All-cause death |  |  |  |  |  |  |
| DI-GM | 1.00 (0.96, 1.04) | 0.973 | 0.91 (0.87, 0.94) | <.001 | 0.92 (0.88, 0.95) | <.001 |
| DI-GM (Quartile) |  |  |  |  |  |  |
| Q 1 | 1.00 (Reference) |  | 1.00 (Reference) |  | 1.00 (Reference) |  |
| Q 2 | 1.01 (0.83, 1.22) | 0.937 | 0.96 (0.80, 1.17) | 0.716 | 0.97 (0.80, 1.17) | 0.729 |
| Q 3 | 0.94 (0.78, 1.14) | 0.547 | 0.81 (0.67, 0.98) | 0.034 | 0.78 (0.64, 0.94) | 0.010 |
| Q 4 | 1.04 (0.87, 1.24) | 0.686 | 0.71 (0.60, 0.85) | <.001 | 0.74 (0.62, 0.88) | <.001 |
| *P* for Trend | 0.723 | | <.001 | | <.001 | |
| BGMS | 0.95 (0.90, 0.99) | 0.033 | 0.86 (0.82, 0.91) | <.001 | 0.88 (0.84, 0.93) | <.001 |
| UGMS | 1.07 (1.02, 1.14) | 0.013 | 0.99 (0.93, 1.05) | 0.680 | 0.98 (0.92, 1.04) | 0.516 |
|  |  |  |  |  |  |  |
| Cardiovascular death |  |  |  |  |  |  |
| DI-GM | 0.95 (0.88, 1.02) | 0.165 | 0.86 (0.80, 0.93) | <.001 | 0.87 (0.80, 0.94) | <.001 |
| DI-GM (Quartile) |  |  |  |  |  |  |
| Q 1 | 1.00 (Reference) |  | 1.00 (Reference) |  | 1.00 (Reference) |  |
| Q 2 | 1.03 (0.72, 1.46) | 0.880 | 0.99 (0.69, 1.41) | 0.938 | 1.01 (0.70, 1.44) | 0.974 |
| Q 3 | 0.94 (0.66, 1.33) | 0.713 | 0.81 (0.57, 1.16) | 0.253 | 0.78 (0.55, 1.12) | 0.176 |
| Q 4 | 0.86 (0.61, 1.20) | 0.371 | 0.59 (0.42, 0.83) | 0.002 | 0.61 (0.43, 0.86) | 0.005 |
| *P* for Trend | 0.244 | | <.001 | | <.001 | |
| BGMS | 0.92 (0.83, 1.01) | 0.071 | 0.83 (0.76, 0.92) | <.001 | 0.86 (0.78, 0.95) | 0.002 |
| UGMS | 1.00 (0.90, 1.12) | 0.937 | 0.92 (0.82, 1.02) | 0.125 | 0.91 (0.81, 1.01) | 0.086 |

HR: Risk Ratio, CI: Confidence Interval

Model 1, unadjusted;

Model 2, adjusted according to age, gender and race;

Model 3,adjusted according to age, gender, race, educational attainment, marital status, BMI, PIR, hypertension, drinking, smoking, physical activity, coronary heart disease, stroke, cancer, insulin use status and total cholesterol.
